# Structure Based Descriptors for the Estimation of Colloidal Interactions and Protein Aggregation Propensities

**DOI:** 10.1371/journal.pone.0059797

**Published:** 2013-04-02

**Authors:** Michael Brunsteiner, Michaela Flock, Bernd Nidetzky

**Affiliations:** 1 Research Center Pharmaceutical Engineering, Graz, Austria; 2 Institute of Inorganic Chemistry, Graz University of Technology, Graz, Austria; 3 Institute of Biotechnology and Biochemical Engineering, Graz University of Technology, Graz, Austria; Instituto de Tecnologica Química e Biológica, UNL, Portugal

## Abstract

The control of protein aggregation is an important requirement in the development of bio-pharmaceutical formulations. Here a simple protein model is proposed that was used in molecular dynamics simulations to obtain a quantitative assessment of the relative contributions of proteins’ net-charges, dipole-moments, and the size of hydrophobic or charged surface patches to their colloidal interactions. The results demonstrate that the strength of these interactions correlate with net-charge and dipole moment. Variation of both these descriptors within ranges typical for globular proteins have a comparable effect. By comparison no clear trends can be observed upon varying the size of hydrophobic or charged patches while keeping the other parameters constant. The results are discussed in the context of experimental literature data on protein aggregation. They provide a clear guide line for the development of improved algorithms for the prediction of aggregation propensities.

## Introduction

The control of protein aggregation in the context of formulation development for biopharmaceuticals is a field of research with growing importance as the number of new bio-pharmaceutical drug candidates in various stages of development is steadily increasing. [1], [2] The required high protein concentrations and the often complex composition of drug formulations can render their development a time and cost-intensive multi-dimensional optimization task. [3], [4] Theoretical models for the prediction of relative protein aggregation propensities based on sequence or structure data would enable a more rational approach and accelerate the development of new or improved drug candidates. A number of such models have been proposed and can be broadly divided into two categories: Statistical approaches and physics based models applying molecular simulation. The former rely to some extent on chemical intuition, using descriptors such as net-charge or sequence patterns, and often include empirical parameters fitted to reproduce experimental data. [5]–[7] The empirical nature of these models can limit their transferability and makes it difficult to quantify the relative contributions of colloidal versus conformational in-stabilities to aggregation. [8], [9] A more serious limitation is the fact that these statistical models were generally trained and tested using experimental data on amyloidosis only. [10] Thus they are unlikely to be applicable to a broader class of protein aggregation phenomena. Physics based approaches can, in principle, predict the contribution of colloidal interactions to aggregation propensities. [11]–[13] Here a difficulty is the complexity of such systems and the involved time and length scales, which require an exorbitant computational effort. A reduction of this effort through the introduction of simplifications such as coarse grained protein models and an implicit solvent representation, limits the accuracy of such approaches. [13]–[15].

An interesting semi-empirical approach is the calculation of spatial aggregation propensities (SAP) as proposed by Trout and co-workers. [16] The SAP is a structure based descriptor for the hydrophobicity of patches on a proteins surface which the authors use as a measure for aggregation propensity. Recently this concept was extended resulting in a new descriptor called developability index (DI), [17] a linear combination of the proteins SAP and its net-charge. Calculation of the DI requires as input merely the proteins structure, or, if a homology model can be used, the protein sequence. This and the fact that its calculation involves a comparatively small computational effort makes it an attractive tool for a quick in-silico pre-screening of protein aggregation propensities. However, so far the predictive power of this descriptor has not been demonstrated beyond the a small number of cases discussed in the original publication. Also experimental results have been published which suggest that, at least in some cases, hydrophobicity and net-charge are not the dominating factors determining aggregation propensities. For example Chari et al. showed that for a human antibody the dependence of protein-protein interactions on the pH can be explained by a combination of the proteins net-charge and dipole moment. [18] Yadav et al. studied the viscosity of concentrated solutions of two anti-bodies and various mutations thereof. [19], [20] They found that the presence of charged, rather than hydrophobic, patches in the proteins complementarity determining regions determine their aggregation propensities. in a recent publication the authors demonstrate that high-concentration viscosity behavior of monoclonal antibody solutions does not show a clear correlation with the proteins’ net-charges. [21] A comprehensive study involving the experimental determination of solubilities of proteins encoded in the EColi genome the authors find no correlation between solubilities and hydrophobic residue content. [22] In a more recent study Price et al. come to a similar conclusion. [23].

Taken together the limited experimental evidence available so far does not allow for a clear conclusion regarding the relative importance of various structure based descriptors such as net-charge, dipole moment, and the presence of hydrophobic or charged patches. The fact that the structures and sequences of the proteins used in many published studies are not in the public domain is not helpful. Even if more experimental data were available it would still be difficult to quantify the relative impact of different properties from experimental data alone since numerous effects and parameters, including the presence of mutations, the buffer type and ionic strength, partial unfolding, the effect of osmolytes, etc, can be inter-dependent and in an experimental setup it is difficult or impossible to control all these parameters independently.

To summarize, the available experimental data is difficult to compare and interpret. Here we address this issue and our results contribute to the understanding of the relative impact of a proteins physico-chemical properties on its colloidal interactions. The basis of the present study is a simple protein model that is used in Molecular Dynamics (MD) simulations. The model features physico-chemical descriptors with values inside the ranges typically found in small globular proteins. The descriptors considered here include the net-charge, the dipole-moment, and the hydrophobicity and polarity of surface patches, respectively. Using this model we determined the dependence of colloidal protein-protein interactions on each of the four descriptors. We find that variations of the net-charge and the dipole moment, both, have a clear and, importantly, comparable impact on the strength of these interactions. By contrast the variation of protein-protein interactions due to the presence of hydrophobic or charged patches is comparatively small and does not follow a clear trend. We interpret our results in the light of available experimental data and discuss their implications for the development of improved models for the prediction of protein aggregation propensities.

## Results

### The Protein Model

The protein model and the simulation protocol used here were carefully designed to include important factors such as entropic contributions by using an explicit solvent model, to be simple enough to allow for the collection of semi-quantitative results for a range of test cases, and, most importantly, to allow for a clear assignment of effects to causes. Two major differences between this model and existing molecular simulation approaches [13], [15] promise to provide new insights: The solvent is represented by an explicit water model and the protein model is designed in a way that allows for a rigorous assessment of the relative influence of net-charge, dipole-moment and the size of hydrophobic and charged patches on colloidal interactions. For this protein model, in the following referred to as pseudo-proteins (PP), we put forward the following requirements: i) PPs are roughly spherical and fairly rigid to improve the speed of convergence for simulations in explicit water; ii) PPs are large enough to resemble real proteins with respect to the size dependence of the hydrophobic effect; [24], [25] iii) like proteins, the model features a heterogeneous and asymmetric surface charge distribution; iv) PPs can represent molecules with a range of different surface charge distributions, net-charges and dipole-moments, as found for real proteins, and, within certain limits, each of these parameters can be varied independently of the others.

**Figure 1:**
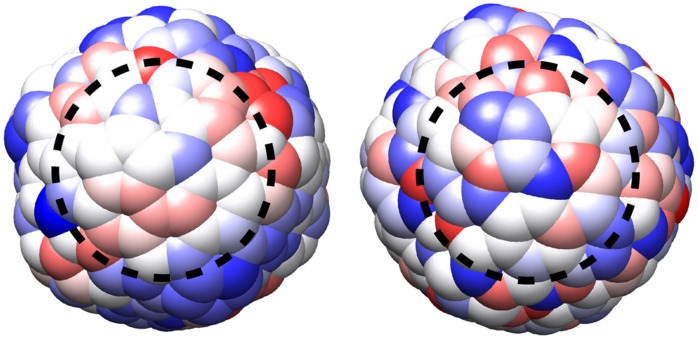
Two PPs with different surface topologies. Left: PP10, right: PP08. Atoms are colored according to the net-charges they carry (blue negative, red positive, white neutral). The PPs are oriented so that the atom with the maximum lssc value, the center of the patch with the highest hydrophobicity, is in the center of each representation.

As a basis for the PP model the structure of a 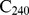 fullerene was used, a molecule that is relatively rigid, roughly spherical, and, with a diameter of about 1.8 nm, of a similar size as small globular proteins or protein domains. One additional atom was positioned at the center of each PP interacting with a steep repulsive potential with the other atoms to better maintain the spherical shape of the PPs. To obtain molecules that resemble proteins partial charges were assigned to each carbon atom. These charges were assigned randomly but biased so as to reproduce, on average, a charge distribution similar to the one found on real proteins. To establish such distributions a set of 55 small globular proteins for which crystal structures are available were analyzed. Charges based on the widely used Amber99 all atom protein force field [26] were assigned to the atoms in each protein and the solvent accessible surface areas (SASA), and the relative frequencies of SASAs of atoms with a particular charge were calculated as histogram. The latter represents a probability distribution of charges, as shown in Figure S1. The charges assigned to the atoms of a PP were then drawn from this distribution and randomly assigned to the atoms on a 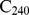. The Van der Waals interactions between the PP atoms and between PP atoms and water were modeled as Buckingham potentials. Initial Buckingham parameters were chosen so that the resulting potentials resembled the Lennard Jones potentials of polar peptide atoms in the Amber99 force field. Both, the charges and the parameters for Van der Waals interactions were further optimized to reproduce average protein-water interactions established beforehand in a set of MD simulations of proteins in explicit solvent. More details about this procedure and the resulting parameters are given in the SI. In the following a given charge distribution on a PP will be referred to as a topology. Examples of two PPs with different topologies are shown in Figure 1.

### Selection of Topologies

In house software was used to generate about 36000 random topologies with protein-like surface charge distributions, as discussed in the previous Section. To allow for a comparison between real proteins and results calculated here PPs with net-charges and dipole moments within appropriate ranges need to be used. To establish such ranges we used a set of models of small globular proteins from the protein data base [27] and assigned partial charges from the Amber99 force field. [26] Details are given in the SI. The resulting ranges are for the net-charge, a valency ranging from 0 to −10, and for the dipole moment a range from 0 to 70 eÅ. Qualitatively, these ranges are confirmed by published experimental and theoretical data. [28], [29].

The charge distribution on a proteins surface can be expected to have an impact on protein-protein interactions, both, through interactions between charged patches, attraction through interactions between regions of charges with opposite or repulsion between regions with equal sign [20], [30], and through contributions from interactions between, and thus the desolvation of, hydrophobic patches. [16] To assess these effects two descriptors are introduced here. The local sum of squared charges (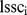) of a region centered on a given atom i on a PPs surface is.
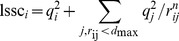
(1)


This is the sum of the squared partial charges on all atoms closer than a certain threshold 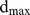 to an atom i. See Figure 1 for a visualization of this concept. The contribution of atoms other than the primary atom i can be scaled by their distance, giving rise to the factor 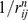. The effect of varying the parameters 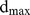
and n in Eqs. 1 and 2 is discussed below. The second descriptor defined here is called local sum of charges (lsc)
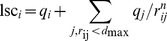
(2)which is the sum of the partial charges on all atoms closer than a certain threshold 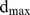 to an atom i. Again the contribution of atoms other than the primary atom i can be scaled through a factor 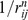.

As a measure for the colloidal interactions, and thus aggregation propensity, between a pair of PPs with a given topology we use mlssc, its smallest lssc value. The smaller this number, the lower the charge density of a patch of a particular size, and the more aggregation prone a particular topology is assumed to be. This assumption is based on the same concept as the SAP. [16] The effect of charged patches is measured by mlsc, the product of the maximum and minimum lsc values for a given topology. For a protein with, both, an extended positively and negatively charged patch we can expect to see attraction, reflected by a large negative mlsc value.

Out of the random topologies three sets were selected. Details are summarized in Table 1. Set varHP: twelve topologies with identical net-charge of −2 e and dipole moment of 18 eÅ but with a range of mlssc and mlsc values. Set varQ: three topologies with varying net-charges. Here one topology of set varH (PP07) was selected and the central atom was assigned a charge of −4 e or −8 e, instead of the original charge of zero, resulting in three topologies with identical values for mlssc and mlsc and dipole moment but net-charges of −2, −6, and −10 e. Set varD: six topologies with a constant net-charge of −2 e and a variation of dipole moments. It turned out being difficult finding topologies with a wide variation of the dipole moment but constant mlssc and mlsc values, thus the latter vary within set varD.

**Table 1:** Properties of pseudo proteins used in this study.

Name	q^a^	P^b^	lssc_min_	1sc_min_	ΔG_min_ c	error d
set varHP						
PP01	−2	18.07	0.59	−6.7	−37.7	5.0
PP02	−2	18.14	0.56	−5.7	−36.1	3.6
PP03	−2	18.04	0.37	−12.2	−34.5	4.9
PP04	−2	17.98	0.58	−14.0	−28.9	4.8
PP05	−2	18.00	0.66	−13.1	−34.1	4.6
PP06	−2	18.32	0.45	−9.2	−36.2	4.2
PP07	−2	17.86	0.59	−4.1	−31.7	1.9
PP08	−2	18.08	0.67	−3.9	−29.4	2.0
PP09	−2	18.15	0.61	−11.5	−17.6	4.5
PP10	−2	18.20	0.31	−8.4	−30.1	3.9
PP11	−2	17.76	0.54	−10.9	−30.8	4.3
PP12	−2	17.92	0.58	−4.8	−28.7	1.6
set varQ						
PP07	−2	17.86	0.59	−4.1	−31.3	1.7
PP13	−6	18.27	0.59	−4.1	−7.7	3.9
PP14	−10	18.71	0.59	−4.1	16.0	1.8
set varD						
PP15	−2	0.65	0.57	−1.9	−31.4	1.4
PP07	−2	17.86	0.59	−4.1	−31.8	1.7
PP16	−2	35.08	0.55	−8.9	−33.9	1.6
PP17	−2	55.53	0.72	−11.5	−36.5	4.1
PP18	−2	65.75	0.80	−17.1	−52.1	5.8
PP19	−2	71.95	0.68	−32.6	−79.0	10.3

a Net-charge in elementary charge units, e.

b Dipole moment in eÅ.

c first energy minimum in potential of mean force in kJ/mol.

d Error bars from boot-strap analysis.

### Effect of n and d
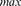


mlssc and mlsc values were calculated for all topologies within set varPH with 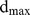 = 2.65 Å and 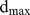 = 5.0 Å. Given the geometry of the PPs a value of 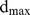 = 2.65 Å ensures that the surface region considered represents a spherical region including 10 atoms. For 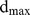 = 5.0 Å this number is 30. In each case results with n = 0, 1, and 2 were compared. It was found that the dependence on the parameter n is relatively weak. Any combination of sets of mlssc and mlsc values calculated at different values of n generally showed a Pearson correlation greater than r = 0.9. Good correlations (between r = 0.78 and 0.99) were also found when comparing mlsc values for the topologies in set varPH calculated at the two values considered for 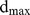. For mlssc values this correlation was comparatively poor, e.g. with n = 0 a correlation of r = 0.32 is found between mlssc values calculated at 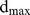 = 2.65 Å and 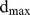 = 5.0 Å. 5 Å is about the largest value for 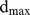
that can be considered here due to the size and convex geometry of the PPs. Also it was suggested that patches around 5 Å are optimal in the calculation of the conceptually similar SAP values. [16] Therefore in the following mlssc and mlsc values with 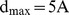 and n = 0 will be reported.

### Free Energies of Association

**Figure 2:**
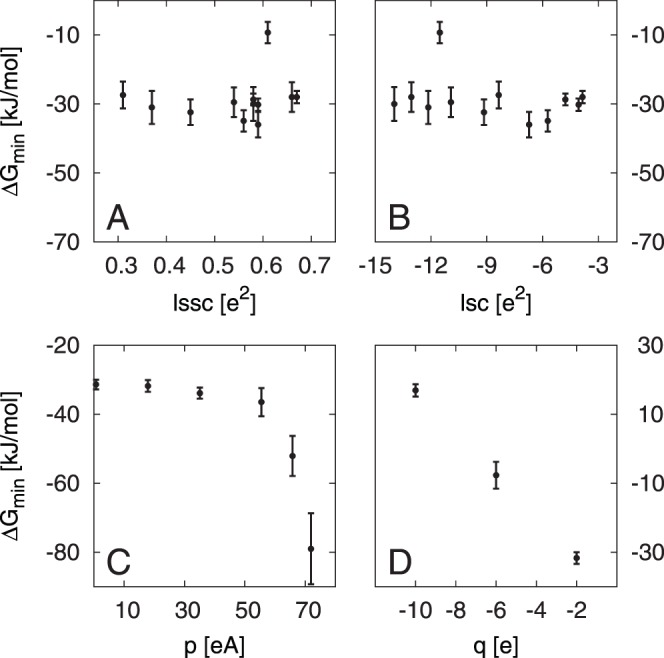
Correlations between the interaction strength and ΔG_min_ descriptors. A: the surface charge variation (SCV); B: a hydrophobicity descriptor (QH); C: the dipole moment p; D: the net-charge q.

**Figure 3:**
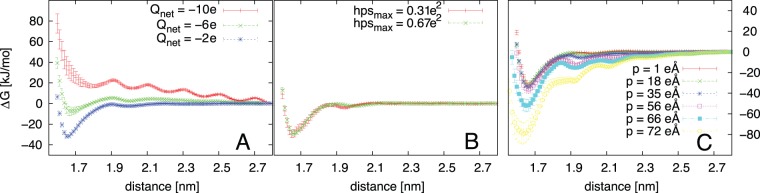
Calculated potentials of mean force between pseudo proteins. A: three PPs with varying net-charge, constant hydrophobicity and dipole moment; B: the two PPs with the largest and smallest hydrophobicities (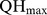) but identical net-charges and dipole moments; C: six PPs with varying dipole moment, identical net-charge and similar 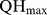
values.

MD simulations as described in the Methods Section were performed for each topology in Table 1. A total simulation time between 360 and 790 nano seconds per topology was required to obtain the numbers given in the following. As a measure for the strength of colloidal interactions the value of the minimum of the potential of mean force (PMF) closest to contact, 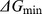
was used here. This corresponds to the free energy of association between two PPs in aqueous solution. The results are given in Table 1. The correlations between 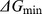
and mlssc or mlsc as shown in the diagrams in panel A and B of Figure 2 demonstrate that 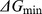
for PPs with equal net-charge and dipole moment does vary to a certain extent but with respect to simple descriptors of the surface charge distribution no clear trends can be observed. This result becomes significant through a comparison with results obtained by varying the dipole-moment or the net-charge as shown in panel C and D of Figure 2 respectively. A clear trend towards greater attraction with decreasing net-charges and increasing dipole-moments can be observed. The differences become clearer when comparing the full PMFs for the topologies in set varQ and varP to those obtained for the two PPs from set varPH with the highest and lowest mlssc values (Figure 3) The difference between the interactions calculated for the latter is negligible compared to the differences resulting from variations of the net-charge and dipole moments. Generally there are, indeed, differences between PPs with varying charge distributions. Calculated values for 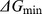
can differ by as much as 18 kJ/mol. However, the two topologies resulting in the least and the most favorable 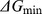
values have nearly identical mlssc or mlsc values. These relations vary somewhat with different values of 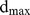
and n but in no case the magnitude of the Pearson correlation between 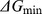
and mlssc or mlsc exceeds a value of r = 0.42. In addition to mlsc we also considered the products of the two highest or the two lowest lsc values of each PP, a measure for the repulsive effect of like-charged patches. Again, in no case the Pearson correlation between these numbers and 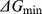
exceeded a value of r = 0.21. The results for set varD, the six PPs with the same net-charge and varying dipole moment, might be attributed to the variations of charged patches, reflected by the variation of mlsc values, rather than just the dipole moments. However, a variation of mlsc values alone cannot account for a substantial variation in 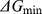, as shown be the results with set varPH. Thus we conclude that the presence of large charged patches can affect protein-protein interactions only if they are accompanied by a substantial variation of the dipole-moment.

Comparison of panel C and D in Figure 2 demonstrates that variations of a molecules dipole moment and net-charge have comparable effects on their colloidal interactions. In order to estimate the practical consequence of this finding we calculated the net-charges and dipole moments for two types of proteins that are typically found in bio-pharmaceuticals, cytokines and antibodies. The proteins are identified by their PDB Ids [27] in Table 2. For each protein Amber99 partial charges, corresponding to a pH of 7, were assigned and the one charged residue whose mutation to a residue with opposite charge leads to the largest reduction of the dipole moment and to an expected increase of the net-charge by two e was determined. The resulting dipole moments and net-charges and the effect of the mutation are given in Table 2. Both, the net-charges and dipole moments of the wild-types exceed the ranges considered for the PPs because the latter are smaller than proteins considered here. However, the relative are more important than the absolute values. Panel C and D in Figure 2 demonstrate that a variation of the dipole moment of approximately 70 eÅ has an effect comparable to a variation of the net-charge by eight elementary charge units. For the proteins in Table 2 the mutation of a single residue can reduce the dipole moment by up to 133 eÅ, on average 37 eÅ for cytokines and 115 eÅ the larger antibodies. At the same time a maximum increase in net-charge of only two elementary charge units can be achieved. These numbers demonstrate that for a given number of mutations the reduction of the dipole moment can lead to a more pronounced effect on colloidal stability than the increase of net-charge.

**Table 2:** Effects of point directed mutagenesis on descriptors.

pdb^a^	mutation	q_0_ b	Δq^c^	p_0_ d	Δp^e^
Cytokines					
1AXI	R134D	−5.0	−2.0	94.8	−43.5
1IL6	R15D	−1.0	−2.0	82.5	−57.0
1RW5	R16D	−3.0	−2.0	99.0	−46.1
1CNT	R189D	−3.0	−2.0	67.8	−48.0
1BGC	R51D	−2.0	−2.0	45.9	−23.5
1F6F	D162R	4.0	2.0	140.5	−53.5
2ILK	D44R	1.0	2.0	154.4	−51.1
1AU1	E107R	4.0	2.0	65.6	−22.2
1BBN	E110R	7.0	2.0	64.2	−27.5
1M4R	E124R	1.0	2.0	85.3	−39.8
1D9C	E13R	8.0	2.0	265.9	−54.8
1LKI	E154R	7.0	2.0	101.6	−50.6
1HUL	E29R	0.0	3.0	72.8	−32.3
1EER	E37R	3.0	2.0	87.4	−36.6
1JLI	E43R	0.0	2.0	47.5	−14.9
1GA3	E58R	3.0	2.0	22.4	−6.8
1EVS	E99R	12.0	2.0	201.5	−56.4
1B5L	K164D	−8.0	−2.0	126.8	−26.0
2HYM	K31D	−2.0	−2.0	114.5	−42.4
1AX8	K5D	−3.0	−2.0	43.7	−10.0
2GMF	K72D	−5.0	−2.0	143.5	−28.7
1IRL	K76D	−0.0	−2.0	101.0	−41.1
Antibodies					
1HZH	D423R	26.0	2.0	345.5	−133.1
1IGT	D31R	5.0	1.0	264.8	−82.3
1IGY	D352R	4.0	0.0	763.2	−128.1

a PDB ID.

b Net-charge of wildtype (WT) in elementary charge units, e.

c Net-charge, difference between mutant and WT.

d Dipole moment of wildtype in eÅ.

e Dipole moment, difference between mutant and WT.

For 22 cytokines and 3 IgG antibodies the variation of net-charge and dipole moment that can be achieved with mutation of a single residue are shown.In three of the 25 cases in Table 2 (1HUL, 1IGT, and 1IGY) the variation of the net-charge caused by the mutation of a charged residue to a residue with opposite sign gave unexpected results, as the magnitude of the resulting charge difference was different from two. Whether this is a genuine effect due to interactions with neighboring residues, or a result of a limited accuracy of the software used here [31] is unclear at this point. However, the answer to this question is beyond the scope of this work, and is not expected to change the overall conclusions.

### Analysis of Literature Data

As discussed in the introduction little is available in the public domain in terms of comparable experimental data for aggregation propensities of different proteins. We found two data sets that seem to be appropriate as test cases for our conclusions. The first, referred to as setA, is a subset of 18 proteins from a large set, comprising a large portion of the EColi strain K12 proteome. [22] The way in which the proteins in this subset were selected is described in the Methods section. The second, setB, is a set comprising the wildtype and 19 single point mutations of RNAseSA. [32] In both cases data for relative solubilities are available. Although aqueous solubility and aggregation propensity are different quantities, we expect to generally find a good correlation between the two. This assumption was recently confirmed for a small set of IgG antibodies. [33].

Structural models for the proteins in the two sets were generated as described in the Methods section and the net-charge, dipole-moment and nSAP, a variation of the original SAP-score, [17] were calculated. Introducing this variation was necessary because in the original form of the developability index the SAP-score was not normalized, presumably because all the protein surface areas in the test set used in the original publication were equal or very similar. To account for the different protein sizes as found in setA we normalized the SAPscores dividing its value, as calculated by the original formula, by the sum of the SASA values of all involved residues, resulting in a value we refer to as nSAP. For completeness we also calculated SAPmax values, the largest of all SAP values for each protein, the number that was used in the original publication that proposed the SAP. [16] The calculated net-charges of each protein where combined with one out of the three descriptors (desc in eqn 3) nSAP, SAPmax, and the dipole-moments to generate three models for the solubility, sol, based on linear regressions (LR), comparable to the developability index. [17].
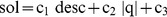
(3)


Recently setA (not the subset used here, but the entire set [22]) was used as a basis for the generation of a model for the sequence based prediction of protein solubilities. [34] This model (CCSol) has been made available online on the web-page of one of the authors. Relative solubilities as predicted by this method (CCSol) were determined for setA and setB using this online server.

The results of this comparison of experimental protein solubilities with various theoretical models are summarized in Table 3. Figures showing details of the statistical analysis that was performed using R [35] are included in (Figure S2). The p-value for a LR model based on net-charge and dipole moment is in both cases smaller than 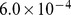, and the correlations between predicted and experimental values in setA and setB are 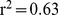 and 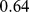, respectively. Models based on a combination of the net-charge and either nSAP or SAPmax generally show poorer statistical significance (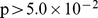) and weaker correlations (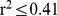). We also tried using the original SAP-score, [17] as opposed to the normalized nSAP (data not shown) but found no improvement of correlations. For no model using p, q or nSAP alone (data not shown) the calculated p-value is better than it is for the model using both p and q. We conclude that for each of the two test sets a statistically significant correlation between protein solubilities and results of a linear regression model based on the proteins net-charge and dipole moment can be found. We also find that, for both protein sets, the a model based on net-charge and dipole moment shows a significantly improved predictive power compared to models based on a combination of net-charge and a descriptor of surface hydrophobicity. Interestingly, if we expand setA, to include, next to proteins that are known to occur as monomers in solution, by additional proteins that are expected to occur as dimers (data not shown) then the correlations between experimental solubilities and the linear regression model of dipole and net-charge are significantly poorer.

**Table 3:** Models for protein solubilities based on molecular descriptors.

model	Regression Coefficients			r^2^	P
	c1	c2	c3		
setA					
LR(q/p)	−0.329	4.339	91.09	0.63	5.82e−04
LR(q/nSAP)	992.9	2.396	31.30	0.33	4.91e−02
LR(q/SAPmax)	7.819	1.714	37.35	0.31	6.37e−02
CCSol				0.43	
setB					
LR(q/p)	−2.671	57.43	226.3	0.62	2.58e−04
LR(q/nSAP)	−1317.5	14.29	51.68	0.41	1.16e−02
LR(q/SAPmax)	−8.403	18.02	33.51	0.29	5.13e−02
CCSol				0.16	

Results from three different linear regression models for protein solubilities, combining the protein net-charge (q) with one of the three descriptors dipole-moment (p), normalized SAP-score (nSAP), or largest SAP value (SAPmax), and from the CCSol web-server. Included are the coefficients of the linear regression models (Eq.3), the correlations between experimental and calculated solubility (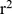), and the P-value (probability that the observed correlation is coincidental). Data are given for two protein sets: 18 proteins from EColi-K12 (setA), and 20 mutations of RNAseSA (setB).

If we are interested in relative, rather than absolute values for solubilities the result of the linear regression can be expressed as a single coefficient, e.g., the ratio between 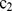 and 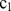 in Eqn 3. For the model based on q and p this ratio is −13.2 (setA) and −21.5 (setB), respectively. Although the two numbers differ they are quite similar considering how different the two data sets are, confirming that in this case q and p are the protein properties dominating their relative aqueous solubilities. Using the SAP based descriptors we not only see much larger differences in this ratio, 0.0024 vs −0.01 for nSAP, and 0.219 vs −2.14 for SAPmax, they even change sign, as for one of the sets (setA) solubility increases, while for the other (setB) solubility decreases with increasing surface hydrophobicity. Interestingly the net-charge/dipole model also out-performs CCSol which is based on a linear regression model using six sequence-based descriptors. [34].

## Discussion

The results shown in Figure 2 and 3 demonstrate that the variation of colloidal interactions due to a variation of hydrophobic or charged patches is less significant than their variation due to changes of the net-charge and dipole moments. The influence of ions which is not considered here will mitigate this effect, but certainly not the overall trends as the ionic strength, I, of bio-pharmaceutical formulations is typically kept relatively low (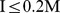), [3] thereby reducing the effect of charge screening. At least for monovalent counter-ions we expect the omission of the effect of counter ions not to effect our major conclusions since the screening of dipole-dipole and charge-charge interactions, and thus their relative contributions to the colloidal stability, will be comparable. Also, the presence of counter-ions can not be expected to result in a trend due to hydrophobic patch sizes when there is no such trend without counter-ions. Counter ions of higher valency can potentially have a pronounced effect on the interactions between charged particles in solution, [36] and even for mono-valent counter ions the protein-ion binding strength can vary considerably depending on the type of ion. [37]–[39] However, the experimental solubility data [22], [32] (setA and setB) analyzed here demonstrates that even in the presence of substantial ionic concentrations protein net-charges and dipole moments remain the two properties that dominate protein-protein interactions in solution. The fact that a model for the estimation of relative protein solubilities including the dipole moment of a single protein as descriptor gives poorer results when proteins are included that are known to occur as dimers in solution further confirms that the dipole-moment is a viable descriptor.

One might argue that the sizes of the error-bars shown here could obfuscate correlations between descriptors of the charge distribution and colloidal interactions. However, given the fact that the production of the current results required approximately 30 years of CPU time on the nodes of a state of the art computer cluster, further reduction of the errors is difficult to achieve, and, more importantly, this can not be expected to change the main conclusion, namely that the variations of net-charges and dipole moments result in clearer and comparable trends, and in more pronounced differences than a variation of the charge distribution.

The fact that, for the PPs considered here, the variation of 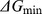
with net-charge is comparable to its variation with the dipole-moment is an important and non-trivial result. In a number of recent studies it was suggested that the protein dipole moment can be instrumental for protein-protein interactions in concentrated solutions, [18]–[20], [40], [41] Our study corroborates these findings, suggesting that the comparable impact of net-charge and dipole moment on colloidal interactions is a general feature of proteins. To our knowledge, the dipole moment has, so far, not been considered as a part of a model for the prediction of protein aggregation propensities, in the spirit of the DI. This might be due to the fact that many existing models were designed to asses effects connected with amyloidosis rather than interactions between globular proteins. [5], [42], [43] Amyloidosis typically involves aggregation of unfolded peptides, which do not have a well defined dipole moment, compared to globular proteins as used in bio-pharmaceutical formulations.

One advantage of considering the dipole moment rather than hydrophobicity in the engineering of proteins through mutations of single residues to reduce colloidal attraction is that, in this case, fewer mutations might be required. This can facilitate formulation development and reduce issues due to immunogenicity. Mutations designed to reduce hydrophobicity typically target hydrophobic, and thus neutral, residues, and can increase the net-charge by one elementary charge unit. Reduction of the dipole moment is most effectively achieved by replacing a charged surface residue by a residue with the opposite charge, and can thereby increase the net-charge by two elementary charge units. The analysis we performed with cytokines and IgG antibodies (Table 2) suggests that in most cases the dipole moment can be reduced considerably with a single mutation. A comparison of panel C and D in Figure 2 suggests that, for a protein with a wildtype featuring a medium to large dipole moment, such a single mutation can have a pronounced effect on the stability, usually larger than the effect achievable by merely increasing the net-charge by one or two elementary charge units.

Obviously the protein model and simulation protocol used here have their limitations. The relatively small size and the purely convex geometry of the PP model limits the maximum hydrophobicity considered to patches with a size of approximately 5 Å radius. This, however, is in part accounted for by the range of net-charges and dipole-moments considered here which were chosen so as to be appropriate for proteins comparable in size to PPs. Also purely hydrophobic patches much larger then those considered here are unlikely to occur on real proteins since even the most hydrophobic residues, such as phenyl alanins are necessarily flanked by backbone atoms, i.e., hydrogen bond donors and acceptors. This is also confirmed by results obtained with SAP values where typically a cut-off radius of 5 Å is used. [16] For a larger protein with several different domains, such as an immunoglobuline, two or more surface patches on different domains could align, leading to cooperative effects that are difficult to predict and assess. Therefore, when dealing with large flexible proteins, our results should be interpreted with some caution, until more experimental evidence is available. The lack of protein flexibility is a principal limitation for which there is no easy remedy. However, it is basis of many simple protein models [11]–[13] and is unlikely to change the principle conclusion regarding the relative influence of the descriptors considered here.

Experimental results in a number of recent publications appear to suggest that protein aggregation propensities can vary significantly with the presence and/or size of hydrophobic patches on proteins. [16], [44], [45] This picture appeals to our chemical intuition, and the fact that, for the model system used here, hydrophobic interactions seem to play a comparatively small role seems counter-intuitive. There is, however, a possible explanation for this seeming discrepancy. The size of hydrophobic patches typically found on proteins might be too small to result in a pronounced effect of hydrophobic interactions on colloidal interactions. Thus, the reduced aggregation rates caused by the removal of hydrophobic patches seen in some experiments might be a consequence of a combination of increased net-charge and increased conformational rather than colloidal stability. It has, in fact, been shown that the removal of hydrophobic patches, guided by calculation of SAP values, can, indeed, increase the thermal, i.e., conformational stability of the proteins. [16] If this gets confirmed by further experimental and theoretical work a change of strategy for the estimation of aggregation propensities in-silico might be due. A descriptor for hydrophobicity, like the SAP [16] might, to some extent, correlate with conformational/thermal stability, but there are other algorithms that were specifically designed to estimate thermal stabilities, [46] and applying such algorithms in a concerted fashion together with descriptors for colloidal stabilities might result in a more reliable prediction. The protein model used here cannot account for conformational changes. Due to its simplicity it does not allow for effects other than colloidal interactions by design, but this is its very strength. It can be used to assess the influence of varying a particular parameter while leaving other parameters constant. Thereby it provides a clear guideline to assist the development of improved models for the estimation of aggregation propensities. A comparable result would be difficult to achieve with a purely experimental setup.

This leaves the question which type of stability, colloidal or conformational, dominates the overall stability of protein solutions, which is an active field of research, and beyond the scope of this work. It has been demonstrated in a number of cases that within different regimes of solution conditions either colloidal or conformational stability can be rate limiting [8], [9], [47], [48] and guidelines have been proposed to discriminate between these regimes. [47].

The results presented here suggest that for the in-silico pre-screening of protein aggregation propensities the most promising route is a combination of the two descriptors net-charge and dipole moment for colloidal stability, and a third descriptor that measures conformational stability. According to our results the inclusion of the dipole moment in a combined descriptor will be more effective than the inclusion of SAP values when colloidal stability is rate limiting. This conclusion is confirmed by an analysis of two literature data sets for protein solubilities we performed.

Especially for large proteins for which experimental structures are not available such as immunoglobulins, accurate determination of net-charge and dipole-moment is not trivial, both in-vitro and in-silico [28], [29], [49]–[51] Thus, for the development of improved algorithms for predicting colloidal interactions and protein aggregation in-silico approaches for the estimation of protein net-charges and dipole moments need to be improved and thoroughly tested. A conclusion regarding the optimization of colloidal repulsion through protein engineering is straight forward: Although the accuracy of in-silico calculations of protein charges and dipole moments might be limited trends can be predicted with some confidence, i.e., it is easy to predict in which direction a particular mutation will change dipole-moment and net-charge. Therefore, when attempting to increase colloidal repulsion between proteins by mutations a replacement of a residue that leads to increased net-charge and reduced dipole moment is not only the most promising strategy, but also is the identification of a good candidate for such a mutation straight forward and easy to implement. Since variations in colloidal interactions achievable by variations of net-charge or dipole moment are comparable in magnitude a variation of both through point directed mutagenesis can be expected to have a pronounced effect, in some cases through a single mutation.

## Methods and Analysis

The PMF, the free energy as a function of the distance between the centers of mass of two PPs, was calculated through MD simulations using the simulation package Gromacs. [52] Unless mentioned explicitly default parameters were used. Two PPs were assigned charges corresponding to a particular topology, positioned with a given center-to-center distance in a rectangular simulation box of dimensions 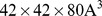 with the line connecting the two centers of mass aligned along the z-axes. The system was solvated with about 4300 TIP3P [53] water molecules. During the MD simulations the central atoms of both PPs were frozen in the directions perpendicular to the z-axes. The length of the simulation cell in the z-dimension, was chosen so that electrostatic long range interactions between proteins in neighboring cells in this direction due to periodic boundary conditions and Ewald summation are small. Thus the system, effectively, corresponds to two isolated molecules in water, and three body interactions are ignored. This is an approximation that is commonly made when calculating potentials of mean force between two molecules in solution. [15], [54].

For the integration of the equations of motion a leap-frog algorithm with a time step of one femto second was used. The temperature was maintained at 300 K using a Nose-Hoover thermostat [55] with a coupling time constant of 0.4 pico seconds. A Berendsen barostat [56] with a coupling time constant of 0.5 pico seconds was used to maintain the pressure at one atm. Here the pressure was controlled by varying only the box dimensions perpendicular to the z-axes in order to avoid interference with the calculation of the forces and distances between PPs. A Particle Mesh Ewald algorithm [57] was used to account for electrostatic long range interactions. The cut-off radius for Van der Waals interactions and the real-space part of the Ewald sum was set to 1.2 nm. During an MD simulation spanning one nano second the distance between the PPs was constrained to the initial value with a harmonic potential acting on the two central atoms, and the force and distance between the two PPs were recorded. This procedure was repeated multiple times for distances varying from 15.5 Å up to 28.0 Å in steps of 0.5 Å. The entire protocol was then repeated at least fourteen times, starting from different initial rotations of the PPs. The latter were produced by generating a large number of conformations with the two PPs randomly rotated around their centers of mass. Only conformations that differed from all other conformations by a minimum difference corresponding to a rotation of one PP through an angle of 90 degrees were kept. In each case the output of the last 500 pico seconds of each MD run was used in the subsequent analysis, while the first 500 pico seconds were discarded as equilibration time. The weighted histogram analysis method [58] (WHAM) as implemented in the Gromacs tool g_wham was used to calculate the PMF for each topology based on the recorded inter particle forces. [58] This program was also used to estimate error-bars through boot-strapping. [59] If for any topology the calculated error-bars of the free energy of adsorption, i.e., the error at the minimum at contact distance, exceeded 5 kJ/mol new starting orientations were generated and more MD simulations performed until the error bar fell below this threshold. Two exceptions (PP18 and PP19 in Table 1) were particularly slow to converge, and here larger error bars were accepted as these do not change any of the conclusions.

For the calculation of descriptors for the proteins in setA and setB a structure for each protein was downloaded from the PDB. [60] If more than one structure was available the X-ray structure with the best resolution (the lowest R-value) was chosen. The mutants of setB were generated using the pdb2 pqr software. [61] Of the original large set of EColi proteins [22] only those proteins were retained which i) have a solved structure in the PDB, ii) have a molecular weight between 15 and 25 kDa, iii) are known to occur as monomers in solution (an information taken from the Uniprot database. [62]) resulting in setA as a small representative test set. For each structure five models were generated by calculating coordinates for any missing atoms or residues with Modeller [63], using default settings. For SetA the C and N-terminal tags present in the proteins used in the experiments [22] were not included when generating the homology models. Since their structures are essentially unknown we expect the tags, if modeled, to introduce more noise than signal to the resulting numbers for charge and dipole. pdb2 pqr [61] and propka [64] were used for the protonation of the resulting structures and for assigning partial charges from the Amber99 force field [26]. For the calculation of solvent accessible surface areas surface_racer [65] was used, and protein net-charges, dipole moments, and SAP-values were calculated using in-house awk scripts.

## Supporting Information

Figure S1 **Distribution of relative surface charges on a sample of 55 cytokine proteins with partial charges and VdW radii from the Amber99 force field.**
(EPS)

Figure S2 **Statistics of linear regression models discussed in the text.** Output of the *summary* command in R.
(EPS)
